# Enriched and Deprived Sensory Experience Induces Structural Changes and Rewires Connectivity during the Postnatal Development of the Brain

**DOI:** 10.1155/2012/305693

**Published:** 2012-07-09

**Authors:** Harkaitz Bengoetxea, Naiara Ortuzar, Susana Bulnes, Irantzu Rico-Barrio, José Vicente Lafuente, Enrike G. Argandoña

**Affiliations:** ^1^Laboratory of Experimental Neuroscience LaNCE, Department of Neuroscience, Faculty of Medicine and Odontology, University of the Basque Country (UPV/EHU), Sarriena Auzoa, 48940 Leioa, Spain; ^2^Unit of Anatomy, Department of Medicine, University of Fribourg, Rue Albert Gockel 1, 1700 Fribourg, Switzerland

## Abstract

During postnatal development, sensory experience modulates cortical development, inducing numerous changes in all of the components of the cortex. Most of the cortical changes thus induced occur during the critical period, when the functional and structural properties of cortical neurons are particularly susceptible to alterations. Although the time course for experience-mediated sensory development is specific for each system, postnatal development acts as a whole, and if one cortical area is deprived of its normal sensory inputs during early stages, it will be reorganized by the nondeprived senses in a process of cross-modal plasticity that not only increases performance in the remaining senses when one is deprived, but also rewires the brain allowing the deprived cortex to process inputs from other senses and cortices, maintaining the modular configuration. This paper summarizes our current understanding of sensory systems development, focused specially in the visual system. It delineates sensory enhancement and sensory deprivation effects at both physiological and anatomical levels and describes the use of enriched environment as a tool to rewire loss of brain areas to enhance other active senses. Finally, strategies to apply restorative features in human-deprived senses are studied, discussing the beneficial and detrimental effects of cross-modal plasticity in prostheses and sensory substitution devices implantation.

## 1. Introduction

After birth, sensory experience modulates the intrinsic developmental programs to shape both functional and anatomical cortical architecture and function from gene expression to activity patterns across systems [1–4]. The postnatal nervous system responds to stimuli from the outside world to develop and consolidate brain connections. Brain circuits are particularly susceptible to these stimuli during a special time window called critical period [3]. After this period, the brain wiring is mature and modifications are more difficult to be made. This natural process can be disturbed by a loss of these stimuli (deprivation) in the different sensory systems, such as visual system [5], somatosensory system [6], or auditory system [7]. Deprivation of sensory inputs throughout postnatal development induces a major disturbance of axonal, dendritic, and synaptic connection patterns of neural circuitry [6, 8, 9]. A major regulator of experience-mediated tuning of sensory systems is the balance between excitation and inhibition [10, 11]. Although the time course for experience-mediated sensory development is specific for each system, postnatal development acts as a whole, and if one cortical area is deprived of its normal sensory inputs during early stages, it will be reorganized by the nondeprived senses in a process of cross-modal plasticity [12, 13]. 

The purpose of this paper is to detail cross-modal effects that can link different sensorial cortices. Particularly review the effects of enriched environment over different senses, specially focused on visual system and the effects of this environment on visually deprived animals. Enriched environment is a tool with neuroprotective effects over many brain diseases and with restorative effects over sensory systems. Through a deeper understanding of this environment, better strategies can be designed to exert cross-modal effects in order to complement the missing sense with the spared ones. 

## 2. Visual System Development

During postnatal development, specific connections among neurons within the visual cortex as well as its inputs and outputs are established, ultimately leading to a functional network. This process is completed in two stages, since environmental experience modulates the genetically predetermined roadmap to shape functional and anatomical cortical architecture and function [2, 3, 14]. Experience exerts effects over the three major elements of the brain; increases the number and size of synapses per neuron [15], the neuronal activity [16, 17], and the metabolic demand [18, 19]; increases astroglial population [20]; causes changes of the vascular network [5, 15, 21].

The structure of the visual system follows the basic outlines of sensory systems. It is a hierarchical system that has a sensitive receptor, some intermediate stations, and a specific area in the cerebral cortex. The fact that it is a pathway known in depth and has great accessibility to each of its components makes it the system of choice in most studies of sensory systems or the cerebral cortex [22]. 

The influence of visual experience begins at eye opening, which in rats occurs in the second week of postnatal life [23]. This is the moment when a major reorganization of all the visual system mediated by visual stimuli begins to be felt; especially during the so-called critical period (maximum period of synaptic reorganization due to experience).

This period exist in many species, from humans to *Drosophila* [24], is specific for each brain area, and after this experience-mediated reorganization of the cortex, the sensory functions reach maturity [3, 25]. The closure of the critical period is completed when anatomical and functional phenomena are established. Structural factors such as perineuronal nets (formed around the neurons) [26, 27] and myelin-related proteins [28] inhibit axonal sprouting. On the other hand, functional changes between excitatory and inhibitory signals, such as intracortical GABAergic inhibition by parvalbumin positive interneurons [29], regulate the termination of the critical period. The exact period of vulnerability for the deprivation of cortical visual stimuli is important for understanding the normal development of the visual cortex. It has been shown that susceptibility to monocular occlusion begins around the end of the third week of postnatal life, peaks during the fourth and fifth weeks of postnatal life, and begins to decline after the end of the fifth week of postnatal life [30].

Although until relatively recent times it was believed that brain lost plasticity after the end of the critical period remaining fixed in adulthood, now it is well accepted that the adult brain maintains certain degree of plasticity to cope with a changing environment throughout life [14], like an extended critical period. Throughout numerous studies, it has been found that a number of interventions can promote plasticity in adult rodents, including environmental enrichment [31], visual deprivation [32], previous monocular deprivation of the same eye [33], enzymatic degradation of the extracellular matrix [26], stimulation of histone acetylation [34], and the antidepressant fluoxetine [35]. 

The best studied model of age-dependent cortical plasticity is ocular dominance (OD), achieved by monocular deprivation (MD). Neurons in the binocular visual cortex respond to inputs from both eyes but are dominated by the contralateral eye (in rodents), and monocular deprivation induces a shift in the ocular dominance of binocular neurons towards the open eye. The ocular dominance is most pronounced in young animals during postnatal development (P25), is reduced in young adults (P95), and is absent in fully mature animals older than 110 days of age [36].

To date, most studies and efforts have focused on young animals, but the studies of the last years have opened a new window for studies of plasticity in adults and their therapeutic application.

## 3. Sensory Deprivation

Modifications of properties of sensory cortices by elimination of its natural sensory inputs (deprivation) serves as a model for studying brain plasticity and his capacity to rewire itself, showing an impressive range of cross-modal plasticity. 

### 3.1. Visual System Deprivation

Although the influence of external experience takes place throughout the central nervous system, most studies on sensory deprivation have been performed on the visual cortex. The absence of visual experience from birth delays normal maturation and maintains the visual cortex in an immature state [20, 30]. In particular, visual connections do not consolidate. They remain plastic well after the closure of the physiological critical period and visual acuity does not develop [26]. The visual system organization facilitates the study of its structures through the interruption of pathways at different stages and through the deprivation of inputs using either invasive methods such as eyelid suturing [30, 37] and unilateral/bilateral enucleation [38], or noninvasive ones, such as dark rearing (DR) [5, 26, 39, 40]. Whereas dark rearing avoids any surgery action and leaves the cortex in an immature state which can be modified by subsequent visual experience [41], eyelid suture requires surgical manipulation and animals receive visual stimulation with diffuse light trough the suture eyelids that result in abnormal binocular interactions in the striate cortex and irreversible development defects on cortical physiology [42]. Enucleation is the most invasive technique that affects either physiologically and at structural level over animals and the effects are not of any greater extent than did dark rearing alone [43]. On the other hand, surgical or invasive techniques allow us to close only one eye so that we can see the effects of monocular deprivation and compensation effects in the contralateral cortex, while dark rearing does not allow this fact, being deprivation bilateral.

Eliminating visual stimulation by dark rearing, alterations at physiological and morphological levels in 3 of the major components of the brain, neurons, astrocytes, and blood vessels are achieved. Deprivation of visual experience reduces synapse-neuron ratio [43] and alters brain-derived neurotrophic factor (BDNF) signaling affecting normal development of visual cortex neurons [44]. The astrocyte population is also affected. Astrocytic density is reduced in visual and somatosensory cortices [20, 40, 45] and the maturation of astrocytes is restricted [46]. Blood vessels, the third element of the neurogliovascular unit, are also affected by the lack of visual stimulation. There is a delay in the maturation of the microvascular pattern of the visual cortex. During postnatal development including the critical period, the vascular density is lower in rats reared in darkness due to decreased synaptic activity and lower energy requirements which need a lower rate of blood supply to meet demand in the cortex [5, 47]. The vascular area was also decreased and the number of neurons is minor, all related to a decrease in cortical activity [48]. The effects on brain vascularization are reflected in the principal angiogenic factor, vascular endothelial growth factor (VEGF). In the precritical period of the rat visual cortex, DR and control animals showed similar VEGF protein values, while during the critical period difference between the two groups were found, characterized by a reduced protein expression translates in a lower vascular density in visually deprived animals [39].

### 3.2. Nonvisual Sensory Deprivation

Another widely used system for sensory deprivation is the somatosensory cortex. Tactile information coming from whiskers plays a key role in the perception of the environment of rodents [6, 49, 50]. A major feature of the rodent primary somatosensorial cortex (S1) is that layer IV contains a unique topographic representation of each facial whisker called a barrel, that is organized forming discrete cytoarchitectonic units [8, 51, 52]. This cortical organization allows the evaluation of the effects of manipulating single whiskers. The whisker map is established during the critical period that extends along the first postnatal week, and therefore precedes the visual or auditory critical periods [53]. Plasticity of the somatosensorial cortex follows the same biochemical pathways of the rest of the senses [53, 54], and the critical period is also characterized by the development, balance, and pruning of excitatory and inhibitory synapses [55]. Impoverishing sensory activity by whisker trimming induces morphological and physiological alterations in the somatosensory barrel cortex when manipulation is performed during the critical period [6, 56–58]. Although the effects and the time course of S1 deprivation on neuronal architecture and function have been widely studied [59], the rest of the elements of the S1 cortex have received much less attention. With regard to vascularization, fMRI studies show a pattern of neurovascular coupling following whisker activity sharing most features of what happens in the visual cortex, where capillary density is higher in the most active areas [60–62]. In an animal model for ischemia, increasing whisker stimulation after a ischemic injury increases the vascularization of the barrel cortex by upregulating angiogenic factors such as VEGF [63], thus, showing similar vascular effects to increased sensorial activity as we previously reported in the visual cortex [39]. 

Studies of other senses have also been mostly focused on neuronal structure and function. Studies on the effects of odor or auditory deprivation share similar effects with the visual or somatosensory systems [6, 64]. Nevertheless, the effects are not restricted to neurons, as olfactory deprivation also reduces the organization of astroglial networks [65].

## 4. Enriched Environment

The first approaches to the effects of environment on development can be traced back to the 19th century with Lamarck or Darwin [66, 67]. The latter reported that rodents raised in nature had bigger brains that caged domestic ones. At the end of the century, both Cajal and Foster advanced the effects of learning on synaptic plasticity [68, 69]. 

The study of experience-induced modification of brain morphology has been performed by conducting studies in a laboratory setting where environmental conditions can be modified [70]. 

**Figure 1:**
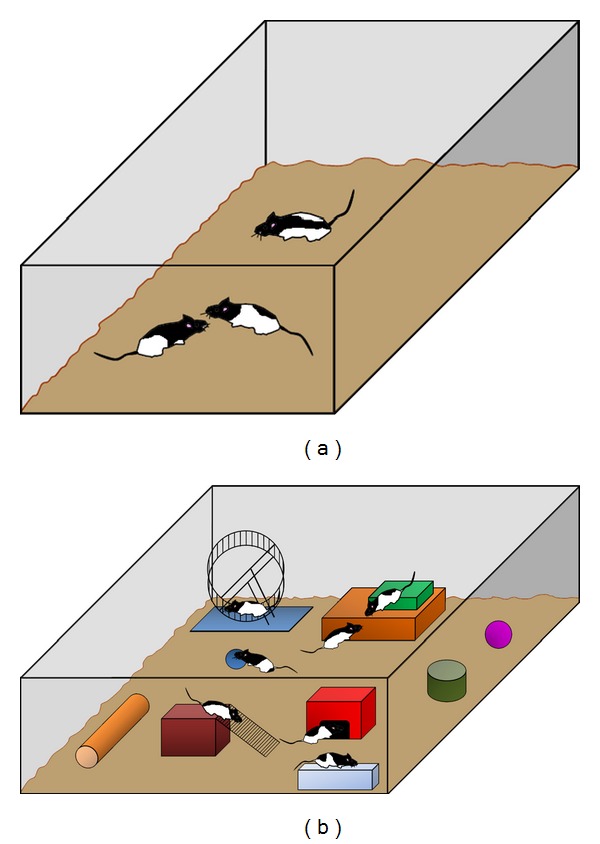
(a) Standard laboratory cage for animal rearing; (b) enriched environment (EE), defined as the combination of complex inanimate and social stimulation, formed by bigger cage than standard ones, full of toys of different colors, shapes, tunnels, material to construct the nest, a shelter, and an exercise wheel.

Although the origin of the studies about effects of environmental enrichment (EE) can be traced back to centuries ago, the first systematic studies can be attributed to Donald Hebb in 1947, when he described how rats taken into his home and cared for as pets performed better on problem-solving tests than rats raised in cages [71]. Rosenzweig, Krech, Bennet, and Diamond, his group of disciples at Berkeley, defined the concept of environmental enrichment as the combination of complex inanimate and social stimulation. From their first studies, the enriched environment has been constantly implemented with cages bigger than standard ones, full of toys of different colors and shapes, tunnels, material to construct the nest, and a shelter, the latter having been recently described as a necessary element of environmental enrichment (Figure 1). These objects have been changed (the best schedule has been established as once every two days) and the placement of food has also been changed on a regular basis. Other elements that have a substantial influence are social interaction, so that wider cages allow rearing a greater number of animals that interchange social stimulation and physical exercise, forced or voluntary, that in rodents is commonly implemented by free access to an exercise wheel or by a treadmill [72, 73]. Some authors doubt whether physical exercise should be included. However, as physical exercise by itself induces brain changes, most enriched environment paradigms, starting from Hebb, have decided to include it.

Environmental enrichment increases sensory, cognitive, and motor stimulation and promotes activation, signaling, and neuronal plasticity in all brain areas, such as sensory ones like visual cortex [74–76], auditory cortex [77], or somatosensory cortex [78] or nonsensory ones like the hippocampus [79], the amygdale [80], the basal ganglia [81, 82], and the cerebellar cortex [83]. 

Enrichment induces effects from cellular, molecular, or genetic levels up to behavioral ones. At anatomical level, initial studies showed that environmental enrichment increases cortical weight and thickness [84, 85]. Posterior works showed that EE increases dendritic branching and length, number of dendritic spines, and size of synapses in some neuronal populations [86, 87]. EE also increases hippocampal neurogenesis, mediated by VEGF [88], inhibits apoptosis [89], and has strong effects on the plasticity of neural connections, especially in the visual cortex [23, 90]. For the rest of the elements of the cortex, similar results have been described. Astrocytic morphology was changed due to exposure to enriched environment [74, 91], and size and density of astrocytes of the visual cortex [92, 93] and the somatosensory cortex were increased [40]. In addition the oligodendroglial density was also increased [92] and the same occurs with vascular density [18, 39, 94]. 

Most of these changes at the cellular level are in concordance with changes in the expression of genes involved in synaptic function and cell plasticity. Enrichment increases the levels of angioneurins, molecules that affect both the neural and the vascular cell processes [95]. Angioneurins include molecules first described as vascular growth factors, such as the archetypal angioneurin VEGF [39] and molecules first described as neurotrophins such as nerve growth factor (NGF) [96], brain-derived neurotrophic factor (BDNF) [90, 97], and neurotrophin-3 (NT-3) [98]. At the same time, it increases the expression of synapse proteins and induces changes in the expression of the subunits of the NMDA and AMPA receptors [99].

Apart from these increases at cellular and molecular levels, recent studies have reported the acceleration of visual system development as a consequence of environmental enrichment. Rearing animals in enriched environments induces earlier eye opening and has electrophysiological effects such as the early development of visual acuity [23].

Last but not least, rearing in enriched environments improves learning and memory [79, 100], decreases cognitive impairment due to aging [101, 102], diminishes anxiety, and increases exploratory activity [103]. Recent studies have outlined the importance of the duration of environmental enrichment, being relevant to the persistence of its effects on behavior [104, 105].

This wide range of effects exerted by EE over the whole brain made this environment a useful tool to improve their effects in brain disorders. Enhanced sensory, cognitive, and physical stimulation was able to mount neuroprotective responses against neurodegenerative processes, traumatic insults, or other forms of adult-onset neural dysfunction. EE delayed onset of cognitive deficits and depression-like behaviors associated with the Huntington's disease (HD) [106], was neuroprotective against rodent neurodegenerative disorder models like Parkinson's disease [107], or was able to ameliorate behavioral abnormalities in rodent models of psychiatric disorders, like schizophrenia [108].

## 5. Recovery from Sensory Deprivation

Brain has a great degree of reorganization following sensory deprivation. A common feature is the compensatory cross-modal plasticity that increases performance in the remaining senses when one is deprived [109, 110]. In sensory plasticity maps, the inputs from deprived senses weaken and shrink whereas spared or enriched signals strengthen and expand [11, 111]. Cross-modal plasticity implies not only physiological changes such as a higher activity of the nondeprived systems, but also the recruitment of the deprived area for the compensatory senses [112].

This process also occurs in nature, as happens in blind rats that have decreased visual areas and expanded the remaining sensory ones [113]. Although cross-modal plasticity plays an important role by compensating a deprived sense, it could notably hinder therapies directed at recovering the deprived sense, as the cortical areas devoted to it have been already “occupied” by nondeprived systems.

**Figure 2:**
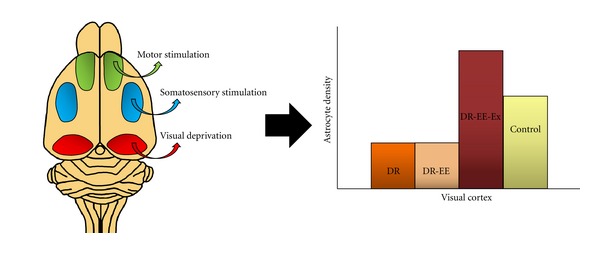
Schematic representation of enriched environment effects on dark-reared rats: motor stimulation, somatosensory stimulation, and visual deprivation caused by dark rearing. We have seen the effects on the astrocyte density of the visual cortex, where enrichment completed with exercise (DR-EE-Ex) can help to recover the loss of population caused by dark rearing (DR), reaching even higher level astrocyte density than the control group. And dark rearing with enriched environment (DR-EE) without running wheel shows similar values to DR group (adapted from results of [40]).

In principle, most of the studies on the recovery of sensorial deprivation have been developed in the visual cortex, but due to cross plasticity, their effects are not restricted to the deprived system, and most of them show effects all over the cortex. Probably the best-known method to compensate sensorial deprivation is by environmental enrichment, also used to compensate the effects of many brain diseases. As previously mentioned, rearing in complete darkness from birth has major effects on the development of the visual cortex, mainly around the critical period. These effects can be altered or modified if animals are dark-reared in complex environments. The first study about prevention of dark rearing effects by environmental enrichment was performed by Bartoletti et al. [104], showing that environmental enrichment promotes consolidation of visual cortical connections, development of visual acuity in dark-reared rats, and restore the effects of dark rearing in chondroitin sulphate proteoglycans developmental organization into perineuronal nets in the visual cortex. In addition to the effects of enrichment on the maturation of cortical connections, effects of enrichment in rats reared in darkness in two of the major elements of the brain, blood vessels, and astrocytes have been analyzed [40, 114]. In our first studies combining this compensatory system, we observed the effects of EE without an exercise wheel in dark-reared rats analyzing the vascular system of the visual cortex. Enrichment cannot recover the deprivation effect over the vascular density of rats reared in darkness. Effects of enrichment, both at the structural level (vascular density) and the molecular level (the level of VEGF protein), were not sufficient to compensate the effects produced by breeding in darkness, and the values of both groups, dark rearing (DR) and dark rearing in enriched environment cages (DR-EE), were similar, lower than the control group [39]. These results remain if we applied the same paradigm to the study of astrocyte density [40]. When exercise is included as part of the enrichment (DR-EE-Ex), recovery effects are observed. DR-EE-Ex environment deprives visual system and enhances somatosensory (darkness force animals to use whiskers to compensate the lack of visual stimulus) and motor systems. The compensatory role existing between different sensory systems is observed. Without visual excitement, an increase in the stimulation of both motor and somatosensorial systems is reflected in the visual cortex, where the density of astrocytes of the DR-EE-Ex group is higher than that of the DR group and the control group (Figure 2) [40].

Another commonly used method to compensate the effects of sensorial deprivation is the exogenous administration of neurotrophins such as BDNF or NGF thus showing their key role in the experience-mediated development of the sensorial systems and controlling the onset and timing of the critical period [115–117]. As exogenous BDNF administration compensates the effects of sensorial deprivation, the previously mentioned effect of exercise on the compensation of visual deprivation could be explained by the fact that exercise upregulates BDNF and thus contributes to the restoration of deprived sensorial systems. In systems other than visual, BDNF administration also compensates at least in part the effects of sensorial deprivation as in the auditory system following deafness [118].

Most of the studies on cross-modal plasticity in humans have been performed in early blind individuals. This process allows us to integrate information received from different senses to elaborate a complex response [119]. In congenitally or early blind individuals, there is an activation of the occipital cortex in response to tactile or auditive inputs that can be demonstrated by an increase in blood-oxygenation-level-dependent responses (BOLD) [120]. Moreover, the visually deprived occipital cortex maintains a high degree of organization and specificity, as the areas that process spatial information in nonblind humans keep this specific function and process spatial information from the auditory system in blinds [121]. Therefore, the spared senses that overtake the cortical area belonging to the deprived one maintain its modular structure. Once the occipital cortex reorganized to process inputs from nonvisual senses, the behavior will be similar. For example, a lesion of the occipital cortex will have effects on the nondeprived sense, such as alexia for Braille in a congenitally blind patient of stroke that mimics the effects of occipital stroke in no-blinds [122].

Apart from visual system, auditory system is one of the mostly deprived systems. Studies have shown that auditory deprivation leads to the recruitment of auditory areas to visual functions [123] or somatosensory functions [124]. In the same way, auditory stimulation activates the visual cortex of early visually deprived anophthalmic mice [125], and bilaterally enucleated rodents have an expanded somatosensorial cortex [126]. 

The cross-modal interactions between somatosensorial and other sensorial systems such as the auditory system also suggest that the effects of recovery strategies for sensorial deprivation are not only circumscribed to the deprived system [59].

Potentially, recovery from auditory deprivation may be closer to practical application in humans than from the other systems. Complete sensorial deprivation is frequent in humans suffering from congenital deafness and as the auditory nerve often is functional, therapies using cochlear implantations have a moderate rate of success, especially if applied before the auditory critical period and if they are combined with auditory enrichment by increasing parent-child interactions [127, 128]. As sometimes happens with handicapped children, the stress produced by a deaf child in hearing parents could lead to an involuntary impoverishing of the language interactions, thus, minimizing the beneficial effects of an early cochlear implantation.

One sense, less studied than the above mentioned is the sense of touch. Body massage and multisensory stimulation are included in neonatal care in human newborns due to their effects on neonates weight gain. In an example of cross-modal effects of early tactile enrichment during development, Guzzetta et al. [129] shows that massage influences the maturation of visual system, accelerating visual acuity development, both in human infants and in rat pups. This effect is exerted by overexpression of IGF in the whole brain. Visual system is not stimulated directly but massage effects have cross-modal effects over the development of this sense.

As cross-modal plasticity could interferes with the recovery of the original function, strategies intended to promote restorative plasticity should also involve the minimizing of interference from cross-modal inputs [130], which could be considered as a side effect of cross-modal plasticity.

Another point of great interest is the development of sensory substitution devices that could convey information of a deprived system (mostly visual) through other sources such as touch or sounds [131]. Cross-modal plasticity is revealed by electrotactile stimulation of the tongue in the congenitally blind [132]. Although the use of neuroprostheses to restore vision in patients has been investigated [133], the results have been far from as convincing as cochlea implants, due to the lack of results and the high degree of invasiveness [134]. Another point of doubt on the use of prostheses is that, as happens with cochlea implants, the occipital cortex has been rewired and inputs from other senses have taken a space. In contrast, sensory substitution devices could benefit from the increasing knowledge of cross-modal plasticity and offer a cheaper, less invasive, and more efficient restorative tool. The fact that recent experimental findings show that the rewired occipital cortex maintains most of the modular features of the nonblind cortex, such as the specialization for spatial processing [121], provides a better substrate to convey complex visual experience from spared senses.

## 6. Conclusions

The lack of sensory inputs from environment during early postnatal development leads to serious consequences that can be reverted reactivating cortical plasticity by physiological strategies such as environmental enrichment, exposure to deprived inputs, or pharmacological solutions as neurotrophin administration. Although the time course of critical period during development is specific for each sensory system, experience-mediated brain development is a unique event. A major feature of sensory deprivation is the cross-modal plasticity process that leads to a compensatory upregulation of the nondeprived senses that even invade the cortical territory of the deprived one. Thus, rehabilitation can be compromised as sensory inputs from the previously deprived sense have to compete with the sensory circuit neoformed by cross-modal plasticity. In conclusion, all restorative strategies against effects of sensory deprivation must take into account that the cortical areas belonging to the deprived sense have a newly established sensory organization that processes inputs from ectopic senses; thus, neuronal segregation is required to ensure that reactivation of the sense is able to form appropriate neuronal circuits. On the other hand, cross-modal plasticity offers a huge opportunity to develop sensory substitution devices that could enable blind individuals to acquire visual information from spared senses as auditory or tactile. The fact that the occipital cortex keeps the functional organization despite the lack of visual inputs, as happens with spatial processing, provides a higher potentiality of visual restoration by nonvisual inputs.
